# Supplementary material for: Validating the indicator “maternal death review coverage” to improve maternal mortality data: A retrospective analysis of district, facility, and individual medical record data
Source: PLoS One. 2024 May 20;19(5):e0303028. doi: 10.1371/journal.pone.0303028 (PMC11104582; doi:10.1371/journal.pone.0303028)
Supplement: S1 File — (DOCX) [file pone.0303028.s001.docx]

| Acuity Label | Definition | Reference |
| --- | --- | --- |
|  |  |  |
| Primary | “Primary health facility” denotes the first level of contact between individuals and families with the health system providing care for mother and child.  May be an ambulatory community health center for ambulatory care or a community hospital with few specialties. Has internal medicine, limited lab services. May be called a community health clinic, primary health center, or a sub-center.  Care of low-risk pregnancies with ability to detect, stabilize, and initiate management of unanticipated maternal-fetal or neonatal problems that occur during the antepartum, or postpartum period until the patient can be transferred to a facility at which specialty maternal care is available.  Care includes non-surgical family planning; immunization, health education, counseling and provision of: basic ANC content; evidence-based management of uncomplicated childbirth; PPFP; well-baby care and feeding. | Adapted from:  Jamison, D. T., J. G. Breman, and A. R. Measham, et al. Disease control priorities in developing countries, 2^nd^ Ed. (2006). Co-publication Oxford University Press, New York, NY and The World Bank, Washington, DC.  Abdul Kareem, PK. 1996. Government of India, Indian Economic Service. Last updated 9 April 2015. <http://www.arthapedia.in/index.php?title=Primary,_Secondary_and_Tertiary_HealthCare>  The Partnership for Maternal, Newborn & Child Health. 2011. A Global Review of the Key  Interventions Related to Reproductive, Maternal, Newborn and Child Health (RMNCH). Geneva, Switzerland: PMNCH.  Kilpatrick, Sarah J., et al. "Obstetric care consensus# 9: levels of maternal care:(replaces obstetric care consensus number 2, February 2015)." American Journal of Obstetrics and Gynecology 221.6 (2019): B19-B30. |
| Secondary | “Secondary health facility” refers to a second tier of health system, in which patients from primary health care are referred to a higher level of care for treatment. May be called a regional, or district, sub-district, block-level hospital.  Provides all services from the Primary level facility plus low-risk intrapartum care and care of appropriate moderate- risk antepartum, intrapartum, or postpartum conditions, i.e., BEmONC signal functions. Every birth attended by at least 1 qualified birthing professional (midwife, family physician, or ob-gyn).  There is an Ob-gyn readily available at all times. Anesthesiology is readily available at all times. An MFM is readily available at all times for consultation onsite, by phone, or by telemedicine, as needed. Internal or family medicine physicians and general surgeons readily available at all times for obstetric patients. Standard obstetric ultrasound imaging with interpretation readily available at all times. |  |
| Tertiary | Highly specialized staff and technological capacity, may be a teaching institution. Has intensive care capacity, cancer, cardiology, specialized imaging equipment. May be called a national, district, provincial, medical college, or academic/teaching hospital.  “Tertiary health facility” refers to a third level of health system, in which specialized consultative care is provided usually on referral from primary and secondary medical care. Specialized Intensive Care Units, advanced diagnostic support services and specialized medical personnel on the key features of tertiary health care.  Provides care of more complex maternal medical conditions, obstetric complications, and fetal conditions, i.e., CEmONC signal functions. Other specialized services include surgical family planning methods, surgical management of PPH, external cephalic version, induction of labor, parenteral antibiotics, manage sepsis, cesarean section.  Ob-Gyn physically present at all times; MFM with inpatient privileges readily available at all times; anesthesiologist physically present at all times. Full complement of subspecialists, such as subspecialists in critical care, general surgery, infectious disease, hematology, cardiology, nephrology, neurology, gastroenterology, internal medicine, behavioral health, and neonatology, readily available for inpatient consultation at all times. Imaging services and interpretation readily available at all times. In-house availability of all blood components. Onsite medical and surgical ICUs. |  |
|  |  |  |
