# Supplementary material for: Validating the indicator “maternal death review coverage” to improve maternal mortality data: A retrospective analysis of district, facility, and individual medical record data
Source: PLoS One. 2024 May 20;19(5):e0303028. doi: 10.1371/journal.pone.0303028 (PMC11104582; doi:10.1371/journal.pone.0303028)
Supplement: S2 File — (DOCX) [file pone.0303028.s002.docx]

| **Supplementary Table 2: Characteristics of Birthing Sites Included in the Study in Argentina, Ghana, and India** |  |  |  |
| --- | --- | --- | --- |
|  | **Argentina** | **Ghana** | **India** |
|  | n (%) | n (%) | n (%) |
| All Facilities | 34 (100.0) | 51 (100.0) | 282 (100.0) |
|  |  |  |  |
| *Setting* |  |  |  |
| Buenos Aires/Bukpurugu Yunyoo/Gonda | 16 (47.0) | 8 (15.7) | 85 (30.1) |
| Jujuy/Sunyani/Krishnagiri | 4 (11.8) | 22 (43.1) | 68 (24.1) |
| La Pampa/Techiman/Meerut | 6 (17.7) | 13 (25.5) | 58 (20.6) |
| Salta/Tolon/Thirvallur | 8 (23.5) | 8 (15.7) | 71 (25.3) |
|  |  |  |  |
| *Facility Type* |  |  |  |
| Primary | 0.0 (0) | 43 (84.2) | 262 (92.9) |
| Secondary | 55.9 (19) | 7 (13.7) | 17 (6.1) |
| Tertiary | 44.2 (15) | 1 (2.0) | 3 (1.1) |
|  |  |  |  |
| *Facility Location* |  |  |  |
| Rural | 0 (0.0) | 16 (31.4) | 241 (85.5) |
| Urban | 34 (100.0) | 35 (68.6) | 41 (14.5) |
|  |  |  |  |
| *Governance* |  |  |  |
| Public | 34 (100.0) | 40 (78.7) | 282 (100.0) |
| Non-profit Private (CHAG) | 0 (0.0) | 1 (2.0) | 0 (0.0) |
| For-profit Private | 0 (0.0) | 10 (19.6) | 0 (0.0) |
| Other | 0 (0.0) | 0 (0.0) | 0 (0.0) |
