# Supplementary material for: Validating the indicator “maternal death review coverage” to improve maternal mortality data: A retrospective analysis of district, facility, and individual medical record data
Source: PLoS One. 2024 May 20;19(5):e0303028. doi: 10.1371/journal.pone.0303028 (PMC11104582; doi:10.1371/journal.pone.0303028)
Supplement: Attachment — Submitted filename: Point by Point Response Table _20230701.docx [file pone.0303028.s003.docx]

| Editor Comments | | Author Response |
| --- | --- | --- |
| Please ensure that your manuscript meets PLOS ONE's style requirements, including those for file naming | | We have ensured that the manuscript meets PLOS ONE’s style requirements |
| Please ensure that you have specified (1) whether consent was informed and (2) what type you obtained (for instance, written or verbal, and if verbal, how it was documented and witnessed). If your study included minors, state whether you obtained consent from parents or guardians. If the need for consent was waived by the ethics committee, please include this information. | | We have added the following: “Patient consent was not required by any of the seven ethical review boards that reviewed the full study methods and instruments because this was retrospective review of health data.”  We note that the full protocol, methods and instruments were reviewed by ethics committees at Harvard and in each study country and none required consent for this retrospective record review. |
| 3. Thank you for stating the following in the Acknowledgments Section of your manuscript:  “This work was supported by the Bill and Melinda Gates Foundation through an award to RRJ and AL (Improving Maternal Health Measurement (IMHM) Capacity and Use, grant number OPP1169546). Funders had no role in study design, data collection and analysis, decision to publish, or preparation of the manuscript.”  We note that you have provided funding information that is not currently declared in your Funding Statement. However, funding information should not appear in the Acknowledgments section or other areas of your manuscript. We will only publish funding information present in the Funding Statement section of the online submission form.  Please remove any funding-related text from the manuscript and let us know how you would like to update your Funding Statement. Currently, your Funding Statement reads as follows:  “This work was supported by the Bill and Melinda Gates Foundation through an award to RRJ and AL (Improving Maternal Health Measurement (IMHM) Capacity and Use, grant number OPP1169546). Funders had no role in study design, data collection and analysis, decision to publish, or preparation of the manuscript.”  Please include your amended statements within your cover letter; we will change the online submission form on your behalf. | | We have removed the discussion of funding from the manuscript file as requested. The Funding Statement as written below should be included in the online submission.  “This work was supported by the Bill and Melinda Gates Foundation through an award to RRJ and AL (Improving Maternal Health Measurement (IMHM) Capacity and Use, grant number OPP1169546). Funders had no role in study design, data collection and analysis, decision to publish, or preparation of the manuscript.” |
| 4. In your Data Availability statement, you have not specified where the minimal data set underlying the results described in your manuscript can be found. PLOS defines a study's minimal data set as the underlying data used to reach the conclusions drawn in the manuscript and any additional data required to replicate the reported study findings in their entirety. All PLOS journals require that the minimal data set be made fully available. For more information about our datapolicy, please see <http://journals.plos.org/plosone/s/data-availability>.  Upon re-submitting your revised manuscript, please upload your study’s minimal underlying data set as either Supporting Information files or to a stable, public repository and include the relevant URLs, DOIs, or accession numbers within your revised cover letter. For a list of acceptable repositories, please see <http://journals.plos.org/plosone/s/data-availability#loc-recommended-repositories>. Any potentially identifying patient information must be fully anonymized.  Important: If there are ethical or legal restrictions to sharing your data publicly, please explain these restrictions in detail. Please see our guidelines for more information on what we consider unacceptable restrictions to publicly sharing data: <http://journals.plos.org/plosone/s/data-availability#loc-unacceptable-data-access-restrictions>. Note that it is not acceptable for the authors to be the sole named individuals responsible for ensuring data access.  We will update your Data Availability statement to reflect the information you provide in your cover letter. | | Our original submission’s data availability statement that was found in the manuscript includes the standard citation and DOI for the publicly available data repository where the underlying data can be found. We have provided the data availability statement as it appears in the original submission below for reference. We will ensure that this statement appears in the online submission system at the time of resubmission as well.  “Data Availability  **All data have been anonymized to ensure compliance with human subject protections and study protocols. The anonymized data underlying the findings are deposited here:**  Jolivet, R Rima; Gausman, Jewel; Adanu, Richard; Bandoh, Delia; Beruetta, Mabel; Chakraborty, Suchandrima; Kenu, Ernest; Khan, Nizamuddin; Odikro, Magdalene; Pingray, Veronica; Ramesh, Sowmya; Vázquez, Paula; Williams, Caitlin; Langer, Ana, 2022, "Validation Data for "Maternal Death Review Coverage"", <https://doi.org/10.7910/DVN/3UEY4I>, Harvard Dataverse, V1, UNF:6:wh1AhrYk9dDrEMsQSRgELQ== [fileUNF]” |
| 5. Your ethics statement should only appear in the Methods section of your manuscript. If your ethics statement is written in any section besides the Methods, please move it to the Methods section and delete it from any other section. Please ensure that your ethics statement is included in your manuscript, as the ethics statement entered into the online submission form will not be published alongside your manuscript. | | We have moved the ethics statement from the Declarations section to the Methods section as requested. |
| Additional Editor Comments:  I concur in large part with the reviewer | | We have addressed all reviewer comments that were specifically mentioned by the editor below. |
| While this has some merit the poor quality of the write up and analysis detract from The findings | We hope that we have adequately responded to the reviewer’s comments. We thank both the editor and the reviewer for their careful critique of our manuscript. | |

| **Reviewer #1 Comments** | **Author Response** |
| --- | --- |
| 1. Is the manuscript technically sound, and do the data support the conclusions?  The manuscript must describe a technically sound piece of scientific research with data that supports the conclusions. Experiments must have been conducted rigorously, with appropriate controls, replication, and sample sizes. The conclusions must be drawn appropriately based on the data presented.  Reviewer #1: Yes | No response needed |
| 2. Has the statistical analysis been performed appropriately and rigorously?  Reviewer #1: Yes | No response needed |
| 3. Have the authors made all data underlying the findings in their manuscript fully available?  The [PLOS Data policy](http://www.plosone.org/static/policies.action#sharing) requires authors to make all data underlying the findings described in their manuscript fully available without restriction, with rare exception (please refer to the Data Availability Statement in the manuscript PDF file). The data should be provided as part of the manuscript or its supporting information, or deposited to a public repository. For example, in addition to summary statistics, the data points behind means, medians and variance measures should be available. If there are restrictions on publicly sharing data—e.g. participant privacy or use of data from a third party—those must be specified.  Reviewer #1: Yes | No response needed |
| 4. Is the manuscript presented in an intelligible fashion and written in standard English?  PLOS ONE does not copyedit accepted manuscripts, so the language in submitted articles must be clear, correct, and unambiguous. Any typographical or grammatical errors should be corrected at revision, so please note any specific errors here. | No response needed |
| 5. Review Comments to the Author  Please use the space provided to explain your answers to the questions above. You may also include additional comments for the author, including concerns about dual publication, research ethics, or publication ethics. (Please upload your review as an attachment if it exceeds 20,000 characters)  Reviewer #1: VALIDATING THE INDICATOR “MATERNAL DEATH REVIEW COVERAGE” TO IMPROVE MATERNAL MORTALITY DATA: A RETROSPECTIVE ANALYSIS OF DISTRICT, FACILITY, AND INDIVIDUAL MEDICAL RECORDDATA  Maternal mortality in LMIC remains is an important topic to explore due to high persistence of MMR even at a health facility level. The authors used an interesting approach to assess the maternal death review coverage. The results of the paper should guide policymarkers on the improvement of maternal mortality data and maternal health programs.  The Introduction is well written. But it is too long. Recommend to shorten it. | We thank the reviewer for their positive feedback about the introduction. We believe it provides critical background information on a highly technical paper; nevertheless, we agree that it was possible to shorten it and have done our best to simplify the language wherever possible to that end. |
| METHOD: Study setting: Even if the authors asked to review the study protocol for more detail, the readers need more information here. Briefly describe why those countries were selected, whether the study is part of a larger study, location of those districts, either rural and / or urban. How the health system is basically organized in each country from lower to higher level health facility, what type of health workers can be found in each level of health facility (for instance: the requirement can be to have at least one nurse and midwife in each health post), etc. | We thank both the editor and reviewer for this comment. We kindly note that this paper appears as part of a PLoS Collection, and we reference our published study protocol that provides extensive background on site selection. We understand, however, that some readers may want more information. In order to make this information more easily accessible for the readers of this manuscript, we have added the following information from the protocol.  “The three countries included in this study were selected through a competitive process with the intention of representing one country from Africa, Asia and Latin America/Caribbean based on World Bank classification. Subnational districts (India and Ghana) and provinces (Argentina) were selected purposefully by way of a two-staged sampling strategy based on a composite index of key maternal health indicators used as a proxy to measure health system performance. One high performing and one low performing state/region in each country were selected for inclusion, as well as one high performing and one low performing district/province within each state were selected. More details on the selection for each study setting are available in the study protocol.”  We have also added the following text, “Eligible facilities were categorized in each country according to primary, secondary, and tertiary, based on a scoping review and synthesis of available facility acuity frameworks, to standardize them across countries. Our facility acuity definitions, with reference citations, are available in Supplementary Table S1” which references the definitions we used for facility levels which include descriptions of their characteristics and capacities, and which were applicable reflections of the health system in each country with regard to maternal health care. We believe describing the health system in general as suggested by the reviewer is too much information and beyond the scope of this study. |
| Data collection and source: Why only public health facilities are included in India and Argentina? What is the data collection period? Tell us more about the expertise data collectors (are they health professionals? How the training was done? Etc.) what tools were used to collect the data, ect… | We have added the following additional information requested per each point above.   1. Clarification on Public/Private facility inclusion by country: We have added the following clarifications, “In Argentina and India, all public facilities where births took place were included in primary data collection. Private facilities were not included in Argentina and India as there were no registered private facilities that reported their data on maternal deaths to the government.” 2. Data collection period: We have added that “Data collection took place from March 2020 to March 2021.” 3. Expertise of data collectors: We have added the following in the manuscript: “The collaborating institutions are each based in one of the countries of study and are comprised of teams that are familiar with the local research context. Further, each institution and research team member have extensive experience in conducting human subjects research in their respective countries. Data collectors in each country were comprised of clinicians and individuals with advanced training in public health.” 4. Training: We have added the following text: “Data collectors underwent extensive training prior to data collection. Training in each country included an overview to the study, research regulations and protocol, how to conduct an interview, field work procedures, data quality, and a detailed review of all data collection tools and procedures.” 5. Tools for data collection: We provide extensive information about the variables that were collected at both facility and district levels. Under the heading “*Facility Data*,” we have added the following information: “Data collected from facilities at all sites used a standard paper-based data collection form with a separate question for each of the variables identified above. Each maternal death was documented individually on the form. One form was used for each facility.” At district/provincial level, we have added the following additional information: “Data extracted from the HMIS system were recorded on a standard paper-based form. One form was used for each facility so that it could be easily matched with the data extracted from facility-records. Further, we have added the following section: “*Data Management and Quality,”* which contains the following additional information *“*All data were entered into RedCap according to a standardized format. All data that were entered into RedCap underwent extensive review by supervisory members of each the study team to ensure accuracy. Study data were managed using REDCap electronic data capture tools hosted at the Harvard Medical School (1, 2). REDCap (Research Electronic Data Capture) is a secure, web-based software platform designed to support data capture for research studies, providing 1) an intuitive interface for validated data capture; 2) audit trails for tracking data manipulation and export procedures; 3) automated export procedures for seamless data downloads to common statistical packages; and 4) procedures for data integration and interoperability with external sources.” |
| District/provincial data: Was individual level data or aggregate level data extracted from HMIS? - If aggregate level, how the linkage was done with facility level data? - If individual level, what variables were extracted to be used to link with facility data (only date of death is not sufficient in my view). You need to convince why only this information was enough to link facility and district level data. Please be more precise. | We thank the reviewer for this comment and we apologize this was not clear in the original submission. We have provided additional information on both the data we extracted from HMIS as well as the linkage process as below under the “Analysis” section of the manuscript. We hope this helps clarify the reviewer’s questions.    With regard to the level of data extracted from the HMIS, data were extracted at the individual death level for each health facility. We have added the following to the manuscript for clarity: “Data were extracted from HMIS on each maternal death that was reported, the facility identifiers where each death was recorded as occurring, and the date of each death. Further, data were collected on whether a death had a corresponding death review reported and the type of documentation (meeting minutes, meeting report, or other) that was provided in support of review.”  With regard to the linkage procedure, we have provided the following additional information: “To do this, we traced individual deaths from the facility by date of death and facility identifiers, including facility name and location, to match the facility record with data on individual deaths extracted from the HMIS. As maternal death is a relatively rare outcome, there were no facilities in any of the three countries that had more than one maternal death occur on a given date. The matching procedure enabled us to assess accuracy of the reported data in the event of a mismatch between the date recorded at a facility and the date recorded in the HMIS. Any mismatches found in the date of death between individual facility records and what was recorded in the HMIS were considered an important finding that enabled us to assess reporting accuracy. Any such cases were further verified and investigated by the research team at the facility and district level.” |
| Data analysis: You need to define your indicators (completeness, content, death with evidence, etc.). What criteria was used for each indicator? For instance, what is “death evidenced at facility” what criteria was used to help classify it as Yes or No. What is completeness of death review, what is the criteria? You may have mentioned it in the Box 1 (Introduction), but we still need it here. | We note that the reviewer's query is really two-fold. We understand it to be related to both our description of the data collected and also variable definitions. We have added the following content in the subsection “Facility Data” under “Data Collection and Sources” to make our definitions clearer.   - For evidence of a maternal death, our original submission stated that “All birthing sites were visited by a trained team of researchers who reviewed medical charts and patient records, hospital registers, and maternal morbidity and mortality review committee reports and records to determine the number of deaths evidenced at the facility during the previous 12 months and to document the completeness of death reviews.” For clarity, we have added the following: “If a maternal death was documented in any of these sources, we recorded it as providing evidence of a maternal death at a facility.” - For evidence of maternal death reviews, our original submission stated that “In all countries, facility data collected on death reviews included death date, review date, type of documentation that supported the review (meeting minutes, meeting report, or other), and content of the review in order to assess the review’s completeness (see Box 1).” To respond to the reviewer’s question, we have added the following: “If a maternal death was documented in any of these sources, we recorded it as providing evidence of a maternal death at a facility.” - We believe that the reviewer is referring to the content of death reviews defined in Box 1 when referencing “completeness,” “content,” etc. This box provides the global definitional standard for content of death reviews and we referenced it in our description of the data collected at facilities. We have made the following revision “In all countries, facility data collected on death reviews in evidence included death date, review date, and type of documentation that supported the review (meeting minutes, meeting report, or other). We further reviewed the content and recorded whether the documentation available substantiated that the review satisfied the seven criteria outlined in the definitional standard (see Box 1). We sought to identify and use the most universally applicable and universally recognized reference standards available. Content was reviewed for compliance with the WHO technical guideline, this study’s reference standard. (3-5)” - For facility type, we have added the following text. “Eligible facilities were categorized in each country according to primary, secondary, and tertiary to standardize them across countries. Our facility acuity definitions are available in Supplementary Table S1.”   We have also added a subsection in the “Data Analysis” section to entitled “Outcome Definitions” for variables we defined at the point of analysis. This applies to the completeness of death reviews. We have added the following language to this section to describe how we defined variables.  “We coded a death review as being complete if it satisfied each of the seven criteria defined in the MPSDR standards for complete maternal death review outlined in Box 1. If it did not satisfy all seven criteria, then it was considered an incomplete review.**”** |
|  |  |
| Socio-demographic characteristics of women (age, place of residence and timing of death): Why only those indicators are selected. Is it based on literature review? How are they selected? Ar those only available variables collected in deathcertificate? Why not include additional variables such as profession, education, marital status, etc… if available. | The rationale for including the socio-demographic variables we selected was described in detail in the previously published study protocol that was cited in the original manuscript. To make this background more readily accessible to the readers, we have added in additional detail as follows:  “The socio-demographic variables included in the study were based on the EPMM Equity Stratifiers and described in the study’s published protocol. (16, 32). These variables were selected because they are routinely available in clinic records in cases of maternal death, and allowed us to disaggregate our data by specific subpopulations facing social disadvantage. Other variables that would allow us to assess equity and discrimination, such as the woman’s educational attainment, wealth, and profession, were not available in retrospective clinical data.” |
| Lines 208-211: We need more details about the linkage between facility and district level data. Variables used for this linkage. I do not think only date of death is enough to link two datasets. There are linkage procedures that were developed to link two datasets using names, place of death, date of death, etc. Be more specific about technic you have used here. | Again, we thank the reviewer for this comment. We have added more information on the linking procedure as described above and the additional variables used in the process as follows:  “To verify the denominator, we compiled the number of deaths that occurred at a facility found through record review and compared it to deaths reported to the district or provincial level in the HMIS. To do this, we traced individual deaths from the facility by date of death and facility identifiers, including facility name and location, to match data on individual deaths extracted from the HMIS. As maternal death is a relatively rare outcome, there were no facilities in any of the three countries that had more than one maternal death occur on a given date. The matching procedure enabled us to assess accuracy of the reported data in the event of a mismatch between the date recorded at a facility and the date recorded in the HMIS. Any mismatches found in the date of death between individual facility records and what was recorded in the HMIS were considered an important finding that enabled us to assess reporting accuracy. Any such cases were further verified and investigated by the research team at the facility and district level.” |
| RESULTS: There are too many tables and figures. Many of them can be combined or summarized. Readers may be lost here with too many repeated results. For instance the last table (table 6), almost all information were found in table 1. | We believe that making our underlying data clear is critical for transparency in our calculations given that this paper is meant to validate an indicator, thus some repetition is expected. That said, we have streamlined the data we presented, revised subheadings, improved table titles, reworked some tables, and removed Fig 2 from the manuscript.  The tables are now labeled as follows:  Table 1: Characteristics of Birthing Sites Included in the Study in Argentina, Ghana, and India  Table 2: Number of Maternal Deaths that Occurred in Facilities and Distribution by Location and Facility Type in Argentina, Ghana and India  Table 3: The Distribution of Maternal Deaths with no review, some evidence of a review, and complete reviews by facility location and type in Argentina, Ghana, and India. Note: we have substantially revised this table to make it clearer for the reader. We hope the reviewer will agree that these edits improve the table’s readability.  Table 4: Sociodemographic Characteristics of Women with Complete vs. Incomplete Death Reviews in Argentina, Ghana, and India  Table 5: Maternal Deaths and Maternal Death Reviews Reported to the District-level from facilities in Argentina, Ghana and India  Table 6: “Maternal Death Review Coverage” Indicator Calculations by Country. Please note: while we wanted to ensure that our underlying data were clear to the readers, we acknowledge that some of the underlying data in Table 6 can be obtained from tables elsewhere in the manuscript. Therefore, we have taken the reviewer’s suggestion and removed those columns from the table. However, as the purpose of the manuscript is to walk the reader through the global indicator calculation, we believe that the calculations in Table 6 are fundamental to this paper. |
| I suggest reorganizing the results’ section to summarize your main findings in 3-4 tables and 1-2 graphs. Numbers from one table to another are different and confusing. | We thank the reviewer for this suggestion. We have removed one table. Our total table count is 6. We have removed one figure so the total number of figures is now 2.  We hope that by including subheadings and streamlining the data presented in the tables that the reviewer will agree with us that the results section is clearer. We also note that the reviewer’s suggestions for including only 3-4 tables and 1-2 charts is not a requirement by the journal, rather a preference.  We are not clear what the author is referring to by stating that the numbers from one table to another are different. |
| Lines 241-258: This section is difficult to read. You start with age group then residence area and talk again about age group. I suggest to reorganize it taking into account one characteristic at a time. | We thank the reviewer for this suggestion. We had previously organized our findings in this paragraph by country rather than characteristic. We have reorganized the paragraph to follow the reviewer’s recommendations. |
| Minors Line 49: correct “indictor” to “indicator” | Done |
| Line 113-5: something is missing in this sentence, not clear | We have revised this sentence. |
| Lines 139-9: Not clear, do you say that death that occurred outside the health facility may be counted in the denominator. I thought this indicator only considers intra-facility (hospital) death. | We have resolved this error which was the result of a typo. We hope that it is now clear as indeed, the indicator does only consider intra-facility deaths. |
| Table 3. Ghana (Yes, column 6), correct n should be 14 instead of 13 | Corrected |
| Table 4. Review the total number for India. | Corrected |
| Line 278: Review the sentence. I think it should be “higher than” instead of “lower than” | This sentence no longer appears in the revision |
