# Supplementary material for: Validating the indicator “maternal death review coverage” to improve maternal mortality data: A retrospective analysis of district, facility, and individual medical record data
Source: PLoS One. 2024 May 20;19(5):e0303028. doi: 10.1371/journal.pone.0303028 (PMC11104582; doi:10.1371/journal.pone.0303028)
Supplement: Attachment — Submitted filename: Final Point by Point Response to Reviewers 12 Sept 2023.docx [file pone.0303028.s004.docx]

Reviewers' comments:

Reviewer's Responses to Questions

**Comments to the Author**

1. If the authors have adequately addressed your comments raised in a previous round of review and you feel that this manuscript is now acceptable for publication, you may indicate that here to bypass the “Comments to the Author” section, enter your conflict of interest statement in the “Confidential to Editor” section, and submit your "Accept" recommendation.

Reviewer #2: (No Response)

2. Is the manuscript technically sound, and do the data support the conclusions?

The manuscript must describe a technically sound piece of scientific research with data that supports the conclusions. Experiments must have been conducted rigorously, with appropriate controls, replication, and sample sizes. The conclusions must be drawn appropriately based on the data presented.

Reviewer #2: Yes

3. Has the statistical analysis been performed appropriately and rigorously?

Reviewer #2: Yes

4. Have the authors made all data underlying the findings in their manuscript fully available?

The [PLOS Data policy](http://www.plosone.org/static/policies.action#sharing) requires authors to make all data underlying the findings described in their manuscript fully available without restriction, with rare exception (please refer to the Data Availability Statement in the manuscript PDF file). The data should be provided as part of the manuscript or its supporting information, or deposited to a public repository. For example, in addition to summary statistics, the data points behind means, medians and variance measures should be available. If there are restrictions on publicly sharing data—e.g. participant privacy or use of data from a third party—those must be specified.

Reviewer #2: Yes

5. Is the manuscript presented in an intelligible fashion and written in standard English?

PLOS ONE does not copyedit accepted manuscripts, so the language in submitted articles must be clear, correct, and unambiguous. Any typographical or grammatical errors should be corrected at revision, so please note any specific errors here.

Reviewer #2: Yes

6. Review Comments to the Author

Please use the space provided to explain your answers to the questions above. You may also include additional comments for the author, including concerns about dual publication, research ethics, or publication ethics. (Please upload your review as an attachment if it exceeds 20,000 characters)

Reviewer #2: This study addressed an important area od validity and quality of maternal death statistics based on MDR. The study has clear approach relating to the indicators used globally and issues addressing both the numerator and denominator.
However, there are some concerns which need to be addressed.

1. While the authors make enormous efforts in six districts over three countries, at the end the number of deaths which from the core of the analyses are small. 17 in Argentina, 14 in Ghana and 58 in India. The numbers are too small to make generalizations even within the country. One cannot escape a sense that there is over-interpretation based on a small set of data.

**Response: We thank the reviewer for this comment; however, we would like to clarify to the reviewer that our study consists of a census of all maternal deaths that occurred within health facilities within a given geographical unit. We could not have a larger “n” because we included all maternal deaths that occurred within all facilities that report to the government within each geographical unit. We believe that the important takeaway of our study is that even though maternal death is rare and there are relatively few maternal deaths in these geographical areas, we still found important challenges with regard to data quality and completeness. We have made the wording more clear in the methods section to emphasize that we have included all maternal deaths (see for example, the revisions on page 12, line 295-296). It seems that this issue highlighted by the reviewer relates more to concerns over generalizability, rather than inference.**

**We further provide extensive discussion of the limitations of our data in the discussion section. Of note, we would like to highlight for the reviewer the following statement in the discussion section:**

**“This study is subject to several strengths and limitations. A strength of this study is that we verified all maternal deaths, maternal death reviews, and content of reviews by collecting primary data from birth facilities, and we cross-validated facility-level data with data reported to districts. In terms of limitations, the number of deaths included in our studies was quite small as maternal death is a rare event. Therefore, our ability to draw statistical inferences from our data is limited and we report numerical, qualitative differences only.”**

**To which we have added based on the reviewer’s feedback:**

**“We can also only generalize to the geographic units in which our study took place, as national-level generalizability is not the purpose of this study.”**

2. While the introduction makes a good case for the need for the study, methods section is quite confusing. Overall, the paper fails to have an engaging narrative and that subtracts significantly from the importance of the paper. I would recommend a tabular approach to list the key questions /indicators and methodological approach to answering them.

**Response: We thank the reviewer for this feedback. We have made extensive revisions to the methods section to more clearly articulate the different ways we operationalized the numerator and denominator. We would like to point the reviewer to the following changes:**

**With regard to the numerator:**

**“To validate the indicator, we varied the indicator’s numerator and denominator and explored the differences in the indicator’s value.**

**We began by validating the numerator’s indicator, which was defined as the number of maternal deaths that occurred at a facility that were reviewed. To validate the numerator, we operationalized it in three different ways using different sources of data:1) the number of maternal deaths that occurred in a facility and that had evidence of a review, 2) the number of maternal deaths that occurred in a facility and that had a review that met the WHO MPDSR standard, and 3) the number of maternal deaths with reviews that were reported to the district/provincial level.”**

**With regard to the denominator, we have added:**

**“The indicator’s denominator is defined as all maternal deaths that occurred in a facility within a given geographical unit. To verify the denominator, we varied the data source from which the counts of facility-based maternal deaths were obtained. We derived the count of maternal deaths from two different sources 1) the total number of maternal deaths evidenced at health facilities, and 2) the total number maternal deaths reported to the district/provincial level.”**

**And finally, with regard to the overall construction of the indicator:**

**“In the final step, we varied construction of the indicator to explore differences in its value. We calculated the indicator three different ways reflecting the different data sources available for both the numerator and denominator as detailed in Table 1.”**

**Table 1: Indicator Definitions for “Maternal Death Review Coverage”**

|  | **Indicator Definition 1** | **Indicator Definition 2** | **Indicator Definition 3** |
| --- | --- | --- | --- |
| **Numerator** | Number of maternal deaths documented at a facility that have evidence of a review | Number of maternal deaths documented at a facility that have evidence of a complete review | Number of maternal death reviews reported to district/province |
| **Denominator** | Number of maternal deaths evidenced at facilities | Number of maternal deaths evidenced at facilities | Number of maternal deaths reported to district/province |
| **Data Source** | Facility | Facility | HMIS |

3. The formation of tables, too many, needs rethinking. Since inter-country comparison is neither attempted nor necessary, could the authors consider using country specific tables with each indicator as a row. and provide a clear narrative of a country as a case study before going to next and maybe use summary table for comparison.

**Response: While we appreciate the reviewer’s suggestion, we respectfully disagree with this comment on the following basis. We believe that presenting each country separately would be repetitive given that there are many shared points derived from the interrogation of each of the indicator’s components across each study area. We think that it is important to highlight the variation in data quality across sites, which would be challenging if we presented as three individual case studies. We systematically interrogate the value of the numerator, then the denominator, then the full value of the indicator across each study.**

4. To fully understand the results and their implications to make the recommendation, it would be useful to add some observations related to the process of reviews and reporting made by the investigators.

**Response: Again, we thank the reviewer for making suggestions to strengthen our paper, but we do not fully understand this comment. If the reviewer is referring to the process for collecting data on the content of death reviews, we respectfully point the reviewer to page 11 where we explain the process for abstracting data on death reviews in detail:**

**“In all countries, facility data collected on death reviews included death date, review date, and type of documentation that supported the review (meeting minutes, meeting report, or other). We further reviewed the content and recorded whether the documentation available substantiated that the review satisfied the seven criteria outlined in the definitional standard (see Box 1). We sought to identify and use the most universally applicable and universally recognized reference standards available. Content was reviewed for compliance with the WHO technical guideline, this study’s reference standard. (9, 12, 35) Data collected from facilities at all sites used a standard paper-based data collection form with a separate question for each of the variables identified above. Each maternal death was documented individually on the form. One form was used for each facility.”**

5. Lack of inclusion of private facilities in India and Argentina is a serious limitation.

**Response: As detailed in the manuscript, we purposefully did not include private facilities in Argentina and India because private facilities in those countries do not report their data to the government, thus there is no HMIS data available for maternal deaths at those facilities. This is detailed on page 10, lines 195-198. We also noted this limitation in the discussion section of the version of the manuscript that was reviewed.**

**To further address the reviewer’s comment, we added the following statement to the limitations noted in our manuscript “In Argentina and India, this is important given that private facilities were not included in the study, which could have an impact on both the numerator and the denominator as death reviews may be less frequent in facilities that are not part of the public hospital network; however, as private facilities in these countries do not report maternal deaths to the government, HMIS data were unavailable from such facilities, which would have limited our ability to compare the value of the indicator using different data sources.”**

Minor:
• Table 3 shows that both in Argentina and India approx. 17% of maternal deaths, had no evidence that a death review occurred (L 325-26); the denominator for Fig1 is not clear, whether it excludes these.

**Response: We thank the reviewer for their close review. We have revised the sentence in the manuscript referring to Fig 1 as follows: “Audit findings detailing the content of each maternal death review conducted by definitional component across study countries are displayed in Fig 1.” Further, we have revised the axis label in the chart to read as follows: “Percent of Death Reviews Conducted Meeting Each Definitional Component.”**

• Table 4 says India complete death review is 48 whereas it is only 24. 48 deaths had any review and not complete review. Needs correction.

**Response: We thank the reviewer for drawing our attention to this error. We have corrected the table.**

• Table 5 can be simplified by deleting all NO as it is a binary and except complicating the table, add no value to the paper.

**Response: We have made the revision suggested by the reviewer and deleted the “no” columns.**

• L204 – six weeks of abortion and 42 days of delivery ).. Aren’t these same then why use different time units.

**Response: We have taken the reviewer’s suggestion and changed 6 weeks to 42 days in relation to abortion for consistency.**

• Table 1 can go as supplementary table.

**Response: We have taken the reviewer’s suggestion and moved Table 1 to supplementary material (now Table S2).**

• Quality of figures are very poor. Fig1 need to have numbers, a horizontal bar could be considered for ease in interpretation. Figure 2 summary is a good idea but was difficult to interpret.

**Response: We are not sure what the reviewer is referring to by mention of a “horizontal bar” or “numbers” in relation to Fig 1, given that it is already presented as a horizontal bar chart. We have added additional tick marks on the horizontal axis in Figure 1 to support interpretation. For Fig 2, the purpose is simply to present and highlight subnational variability. We would like to direct the reviewer to the paragraph on page 27 beginning with line 548 where we provide an in-depth interpretation of the results presented in Fig 2.**

7. PLOS authors have the option to publish the peer review history of their article ([what does this mean?](https://journals.plos.org/plosone/s/editorial-and-peer-review-process#loc-peer-review-history)). If published, this will include your full peer review and any attached files.


If you choose “no”, your identity will remain anonymous but your review may still be made public.


**Do you want your identity to be public for this peer review?** For information about this choice, including consent withdrawal, please see our [Privacy Policy](https://www.plos.org/privacy-policy).

Reviewer #2: **Yes:** Anand Krishnan
