# Supplementary material for: Validating the indicator “maternal death review coverage” to improve maternal mortality data: A retrospective analysis of district, facility, and individual medical record data
Source: PLoS One. 2024 May 20;19(5):e0303028. doi: 10.1371/journal.pone.0303028 (PMC11104582; doi:10.1371/journal.pone.0303028)
Supplement: Attachment — Submitted filename: Response to Reviewers Apr 10 Ind 20.docx [file pone.0303028.s005.docx]

**Response to Reviewers**

Reviewer #2: Overall the paper covers an important area and uses a sound approach.
Abstract:

• Use the term assessed rather than explore the validity of the indicator.

**Response: We changed the term “explored” to “assessed” as follows: “This study assessed the validity of the indicator by examining both its numerator—the number and quality of death reviews—and denominator—the number of facility-based maternal deaths and comparing estimates of the indicator obtained from facility- versus district-level data.” (Page 2, line 51).**

• Add the actual number of deaths covered in the study.

**Response:** We added the following sentence to the abstract “In total, we found 17 deaths in Argentina, 14 deaths in Ghana, and 58 deaths in India evidenced at facilities.”

• Conclusions: First line can be deleted. It says we found incompleteness in deaths recorded. Is this result shown in the abstract?

**Response: We changed the word “incompleteness…” to “we found discrepancies…” This is one of the main findings discussed in the abstract.**

Main Paper:
They selected on high performing and one low performing districts – are they reporting the results by this?

**Response: We included this information for the reader to understand our selection process. We do not present any findings stratified by performance as we think that could be explored in a separate analysis and is beyond the scope of this paper.**

What proportion of the maternal deaths are expected to happen in the private sector needs to be told, even if this is approximate and is from the district level reporting.

**Response: This comment has been discussed extensively in previous versions. We cannot estimate with our data private sector deaths using district level data, as private facilities in India and Argentina do not report deaths to the government. Please see the limitations section for more detail, which reads: ““In Argentina and India, this is important given that private facilities were not included in the study, which could have an impact on both the numerator and the denominator as death reviews may be less frequent in facilities that are not part of the public hospital network; however, as private facilities in these countries do not report maternal deaths to the government, HMIS data were unavailable from such facilities, which would have limited our ability to compare the value of the indicator using different data sources.”**

I still think that there are too many tables and it is best to organize all indicators of one country in a table to give a composite idea of that country to make a meaningful interpretation. Repeated comparisons between countries (which is meaningless) in all tables hinders and is boring beyond a point. The common issues can then be taken up in discussion. The authors feel otherwise. I leave it for the editors to decide.

**Response: We strongly believe that the current format of the manuscript facilitates an iterative interrogation of the value of the indicator and data sources. We have worked extensively on the organization of this manuscript in order to make it clear and succinct. The purpose of the paper is to demonstrate the indicator’s validity in three countries, and it is not possible to pool the data, if that is what is recommended by the reviewer.**
